# Genetic analysis of L123 of the tRNA-mimicking eukaryote release factor eRF1, an amino acid residue critical for discrimination of stop codons

**DOI:** 10.1093/nar/gkv376

**Published:** 2015-04-20

**Authors:** Kazuki Saito, Koichi Ito

**Affiliations:** Department of Medical Genome Sciences, Graduate School of Frontier Sciences, The University of Tokyo, Kashiwa-city, Chiba 277-8562, Japan

## Abstract

In eukaryotes, the tRNA-mimicking polypeptide-chain release factor, eRF1, decodes stop codons on the ribosome in a complex with eRF3; this complex exhibits striking structural similarity to the tRNA–eEF1A–GTP complex. Although amino acid residues or motifs of eRF1 that are critical for stop codon discrimination have been identified, the details of the molecular mechanisms involved in the function of the ribosomal decoding site remain obscure. Here, we report analyses of the position-123 amino acid of eRF1 (L123 in *Saccharomyces cerevisiae* eRF1), a residue that is phylogenetically conserved among species with canonical and variant genetic codes. *In vivo* readthrough efficiency analysis and genetic growth complementation analysis of the residue-123 systematic mutants suggested that this amino acid functions in stop codon discrimination in a manner coupled with eRF3 binding, and distinctive from previously reported adjacent residues. Furthermore, aminoglycoside antibiotic sensitivity analysis and ribosomal docking modeling of eRF1 in a quasi-A/T state suggested a functional interaction between the side chain of L123 and ribosomal residues critical for codon recognition in the decoding site, as a molecular explanation for coupling with eRF3. Our results provide insights into the molecular mechanisms underlying stop codon discrimination by a tRNA-mimicking protein on the ribosome.

## INTRODUCTION

Stop codons are decoded by protein factors called class 1 and class 2 polypeptide-chain release factors (RFs) (1). Class 1 RFs are proteins that functionally and structurally mimic tRNAs, while class 2 RFs are members of the translational GTPase family.

In eubacteria, class 1 RFs enter the ribosomal A site to recognize stop codons and to stimulate hydrolysis of peptidyl-tRNA, thereby releasing the nascent peptide from the ribosome with the aid of the universally conserved Gly-Gly-Gln (GGQ) motif (1). There are two dual-codon-specific class 1 RFs in eubacteria. RF1 recognizes UAA and UAG codons, and RF2 recognizes UAA and UAG codons. The ‘tripeptide anticodon’ of RF1 (P-A/V-T) and RF2 (S-P-F) has been found to be responsible for the discrimination of stop codons (2). Recent studies have revealed the details of the molecular mechanism by which eubacterial stop codon decoding is accomplished; it involves sophisticated interaction of RF1 or RF2 with rRNA as well as with mRNA (3,4). After release of the nascent polypeptide, a class 2 RF, RF3, binds to the ribosome and indirectly facilitates the dissociation of RF1/2 from the ribosome in a GTPase-dependent manner (5).

**Figure 1.**
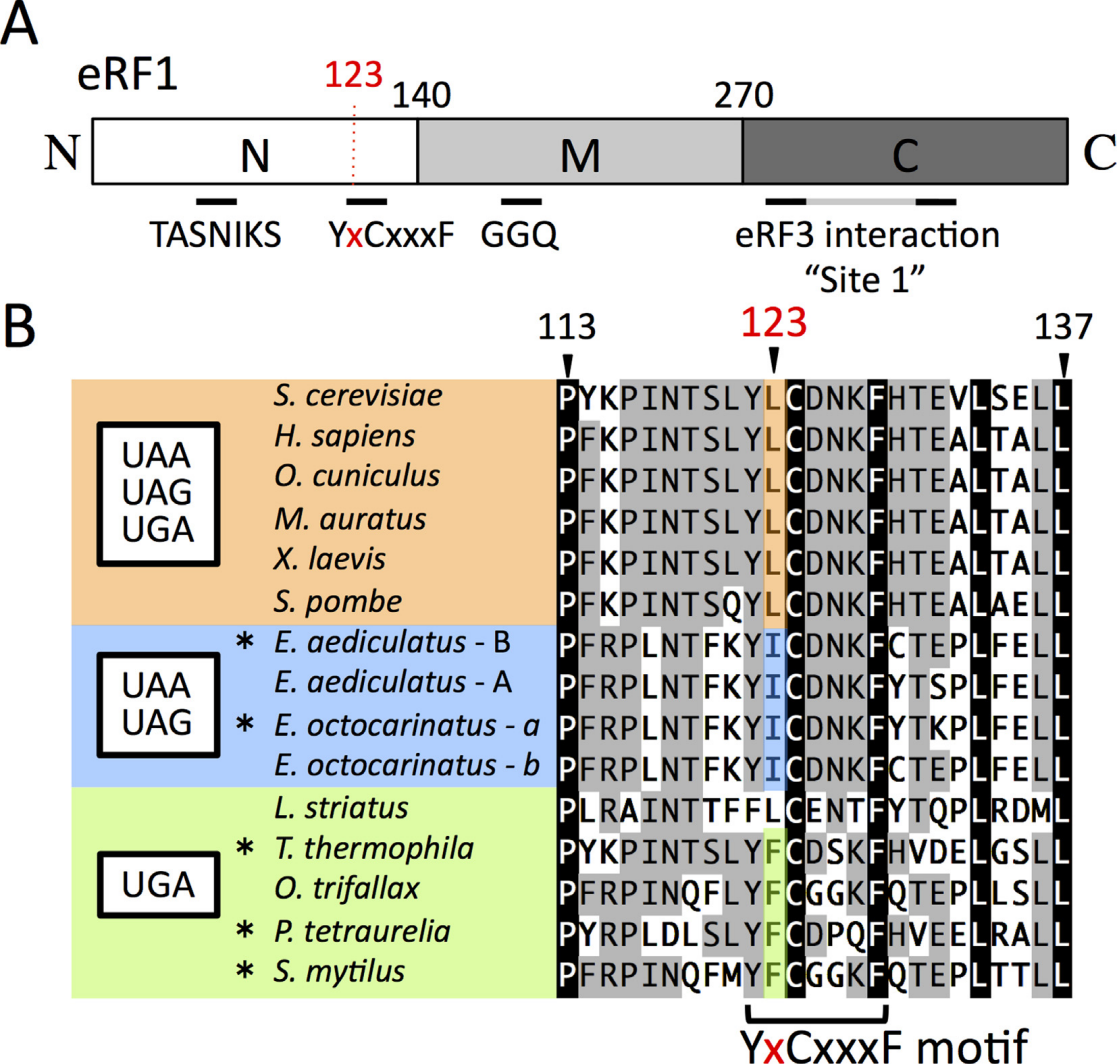
Domains and sequence alignment around L123 of Sc-eRF1. (**A**) Schematic drawing of the domain structure of eRF1. Three domains, according to the structure of eRF1 (domains N, M and C) are shown with the amino acid residue numbers at the domain junctions. Relevant motifs and sites mentioned in this manuscript are also indicated. (**B**) Alignment of eRF1 around the position-123 amino acid residue. Amino acid numbering is based on that of *Saccharomyces cerevisiae* eRF1. Position-123 is shown in red. The YxCxxxF motif is indicated at the bottom. Species with standard and variant stop codon specificity are shown in orange for UAA, UAG and UGA, blue for UAA and UAG and green for UGA. Amino acids at position-123 are highly conserved within each of those categories. The codon specificities of eRF1 indicated here with asterisks (*) have been reported previously (9,27–29) and others are proposed from their codon usage analysis (11,19).

In eukaryotes, the class 1 RF, eRF1, and the class 2 RF, eRF3, are distinct from eubacterial RFs. eRF1 (encoded by *SUP45* in budding yeast) recognizes all three stop codons, i.e. has ‘omnipotent’ recognition, and stimulates hydrolysis of peptidyl tRNA by the GGQ motif (6). eRF1 has three structural domains (Figure 1A) (7). Domain N structurally corresponds to the anticodon stem-loop of tRNA and has been shown to participate in omnipotent stop codon recognition (8). Domain N contains crucial motifs for stop codon discrimination, such as TASNIKS and YxCxxxF (9,10). Domain M contains the universal GGQ motif at the tip of the domain, which is comparable to the CCA terminal of tRNA. Domain C contains the principal site for interaction with eRF3, named ‘site 1’. On the other hand, eRF3 (encoded by *SUP35* in budding yeast) shares high homology with the translational GTPase eEF1A/EF-Tu subfamily (11). Unlike RF3, eRF3 forms a heterodimer complex with eRF1, preferably in the presence of GTP (eRF1–eRF3–GTP complex), prior to entering the ribosomal A site (12), and stimulates peptide release for decoding of stop codons (13,14). This strongly suggests that it is functionally similar to the tRNA–eEF1A–GTP complex for decoding of sense codons.

In Archaea, class 1 RF, aRF1, is highly homologous to eukaryotic eRF1 (15). On the other hand, Archaea do not possess any gene that encodes eRF3 orthologs. Instead, the archaeal EF1A, aEF1A, forms a complex with aRF1 in the presence of GTP and functions in decoding of stop codons (16). In addition, aEF1A also forms a complex with archaeal Pelota (aPelota), which has been suggested to play a role in mRNA surveillance and dissociation of stalled ribosomes (17). Thus, aEF1A is a multifunctional carrier GTPase for adapters involved in elongation, termination and mRNA surveillance in archaea. These findings strongly suggested that the role and the mechanisms by which the translational GTPase decodes sense and stop codons are essentially the same.

Over the last decade, the molecular mechanism by which stop codons are decoded by eRF1 has been studied using numerous approaches, such as bioinformatics (18–20), mutational analyses (8,10,21,22), photo-cross-linking analyses (23–25) and structural analyses (7,26). In particular, functional studies of eRF1s from ciliates that have variant genetic codes, in which one or two stop codons encode amino acid(s), have provided important insights into stop codon discrimination. eRF1s from those ciliates were deficient in conventional stop codon recognition (sub-specific eRF1s) in exchange for the decoding by variant tRNAs (9,27–31). In these studies, crucial amino acid residues and motifs of eRF1 that function in stop codon decoding were investigated. Those amino acids and motifs include GTS (residues 28–30, hereafter indicated in *Saccharomyces cerevisiae* numbering) (20), TASNIKS (residues 55–61) (9), YxCxxxF (residues 122–128) (10), E52 (32), V68 (26) and so on. Some of these amino acid residues are thought to be involved in direct stop codon recognition (8,18,24,26). We have predicted a set of eRF1 residues that are potentially involved in direct stop codon binding, based on the X-ray co-crystal structure of eRF1 with ATP, and mutations at these residues have consistently been found to affect codon specificity (26). However, the precise mechanism by which stop codons are discriminated by eRF1 remains unclear, so that substantial further analyses are required.

Among the amino acid residues, the 123^rd^ amino acid, L123 (according to *S. cerevisiae* numbering), of eRF1, which is located within the YxCxxxF motif, seems to be distinct. In all known X-ray crystal structures of apo-eRF1 (ID: 1DT9) (aRF1 (ID: 3AGK)), as well as of eRF1 in complex with eRF3 (aEF1A) (ID: 3E1Y) (aRF1 (ID: 3VMF) (7,16,26,33), the side chain of the corresponding residues of the 123^rd^ residue lacks intra-domain interactions, and, instead, protrudes from domain N of eRF1 (Supplementary Figure S1) in a direction distinct from that of neighboring residues. Intriguingly, the amino acid conservation at this residue correlates strongly with the variant genetic codes of ciliates (summarized in Figure 1B). Species with a standard genetic code have leucine at this position, while species that encode cysteine by UGA have isoleucine, and species that encode glutamine by UAA and UAG mostly have phenylalanine. Furthermore, single mutations affecting the amino acid at this position in ciliates conferred ciliate-type codon specificities to omnipotent eRF1s (21,22), indicating its phylogenetic and functional significance.

In this report, we focused on the 123^rd^ amino acid residue of eRF1; hereafter, the equivalent residues among orthologs are termed position-123 residues. We conducted systematic mutagenesis of this residue and attempted to elucidate the molecular roles of this residue in codon discrimination by stop codon-specificity analyses *in vivo*. We found that a simple rule applied to sub-specific patterns of position-123 residues as well as that there is a functional coupling of the position-123 residue of eRF1 with eRF3, which was not observed among neighboring residues, suggesting that the position-123 residue belongs to a novel class of functional residues in eRF1 involved in stop codon discrimination. Furthermore, our structural modeling of eRF1 in the quasi-A/T state on the ribosome also suggested a distinctive role for the position-123 residue in the putative molecular interplays on the ribosomal decoding site. We also discuss a model for the putative role of the position-123 residue in stop codon discrimination.

## MATERIALS AND METHODS

### Construction of yeast strains and media

Yeast strains used in this report are listed in Supplementary Table S1. An alternative set of the readthrough assay strains, SKY81, SKY82, SKY83 and SKY84, was constructed from the previously described readthrough assay strains, S13-I01, S13-I03, S13-I05 and S13-I07, respectively (34), by replacing the kanMX selection marker cassette with the CaURA3 gene cassette from pAG60 (35). The readthrough assay strains with the tet-off Sc-eRF3 allele, i.e. SKY106, SKY107, SKY108 and SKY109, were constructed by replacing the endogenous promoters of SUP35 (eRF3) in S13-I01, S13-I03, S13-I05 and S13-I07 with the tet-OFF promoter DNA fragment (36); the kanMX selective marker was replaced with a CaURA3 marker gene cassette flanked by a loxP site and could be removed by Cre recombinase (37). Media for yeast were YPD or synthetic media, prepared with the appropriate dropout mix for plasmid marker selections (ForMedium^TM^; Hunstanton, UK); for plates, 2% agar was added. Paromomycin (Sigma–Aldrich, St. Louis, MO, USA) was added to a final concentration of 10 mg/ml.

### Construction of yeast expression plasmid vectors

The DNA fragments for wild type or mutant eRF1 and eRF3 were amplified by polymerase chain reaction using the DNA primers listed in Supplementary Table S2. The DNA fragments were treated with the restriction enzymes indicated in Supplementary Table S2 and inserted into the compatible cloning sites of the yeast expression vectors that have optimal expression strength and selection markers for each assay (38). Each of the single amino acid mutations was introduced by site-directed mutagenesis using specific primers, as described previously (26).

### Dual luciferase readthrough assay

Readthrough assays were performed as previously described (26,34,39). Yeast transformants were grown in SC-Leu, if necessary, in the presence of paromomycin (10 mg/ml) and/or tetracycline (150 mM). Yeast transformants were grown at 30°C (permissive temperature) in an adequate medium, and then refreshed and incubated at 37°C (non-permissive temperature) for 5 h. Yeast cells were collected by centrifugation and whole cell lysates were prepared by vigorous shaking along with glass beads on the FastPrep 24 instrument (MP Biomedicals, Santa Ana, CA, USA). For measurement of activities of luciferases, the Dual-Luciferase® Reporter Assay System (Promega, Madison, WI, USA) and GloMax^TM^ 96 Microplate Luminometer (Promega) were used according to the manufacturer's instructions.

### Dual eRF1 growth complementation assay with Hs-eRF3

In the Dual-eRF1 assay (26), as a bacterial translation termination system with two codon-specific class I RFs, two eRF1 mutants are co-expressed in a conditional lethal eRF1 strain and the transformant gains viability only when stop codon specificities generated by the combination of eRF1 mutants warrant sufficient activities at all three stop codons in the cell. In this report, an assay strain (Y138) expressing both endogenous eRF1 and eRF3 from the tet-OFF promoter was employed (hereafter called as ‘double tet-OFF strain’) to monitor the combined activities of sub-specific dual-eRF1 mutants introduced by two separate expression vectors (selected by URA3 and LEU2 markers), as well as Hs-eRF3 expressed from an additional vector (selected by a TRP1 marker).

## RESULTS

### Codon-specificity profiles of position-123 systematic mutants of eRF1

A series of yeast expression plasmids encoding *S. cerevisiae* eRF1 (Sc-eRF1) mutants, containing single point mutations of all amino acid residues other than wild-type leucine at position-123, were constructed. The plasmids were introduced into a set of dual-luciferase readthrough assay strains, in which the dual-luciferase construct with either of the three stop codons or the UGG sense codon between the firefly and *Renilla* luciferase was stably integrated into the chromosome. These strains also contained the temperature-sensitive endogenous yeast eRF1, which can be inactivated at the assay temperature (37°C); thus, changes in codon specificities by the mutated eRF1 can be monitored ((26,34,39); see the Materials and Methods section for details).

**Figure 2.**
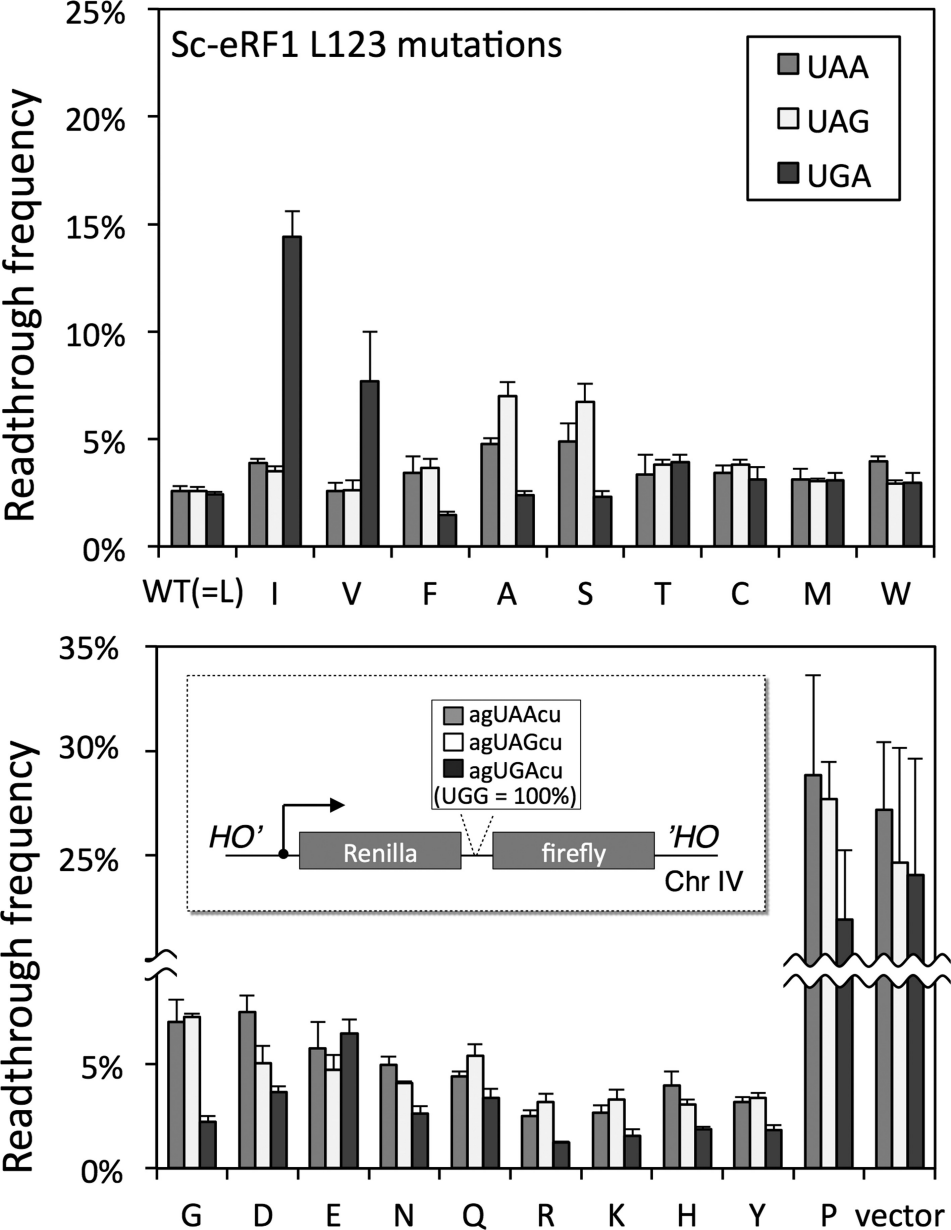
Readthrough assay of systematic Sc-eRF1 L123 mutants. Readthrough frequencies of the systematic substitution mutants of Sc-eRF1 L123 are shown. S13-I01, S13-I03, S13-I05 and S13-I07 (Supplementary Table S1) assay strains that have UAA, UAG and UGA stop codons and UGG codon, respectively, were transformed with the wild type and L123 mutants of Sc-eRF1 on the p416GPD (URA3 marker) expression vector. Readthrough frequencies are indicated by percentage, where 100% readthrough was based on the results of a UGG reporter gene; values are indicated as mean ± SD from three independent measurements (see also Supplementary Table S3). A break in the vertical axis is indicated by a wave-line. Schematic drawing of the dual-luciferase readthrough assay constructs inserted at the *HO* locus of chromosome (Chr) IV of the assay strains is shown within the lower panel. Nucleotide residues for stop codons are shown in capitals and surrounding residues in lowercases.

Intriguingly, the codon-specificity profiles of the position-123 mutations on the stop codons, except for substitution with proline, exhibited only limited patterns of specificities, if any, and the effects could be classified into three groups: ‘unaffected’, i.e. retaining normal omnipotent activity; ‘UAA and UAG dual-specific’, i.e. reduced activity in translation termination at the UGA codon with normal activities on UAA and UAG stop codons; and ‘UGA-specific’, i.e. reduced translation termination at the UAA and UGA codons with normal activities on UGA stop codon (Figure 2 and Supplementary Table S3). Consistently, the second group included L123I and the third group included L123F (Figure 2), as also seen in the ciliate conservation clusters with comparable stop codon specificity (Figure 1B). The fold-change in readthrough caused by the L123I mutation to wild type was 1.5 at UAA (=3.9%/2.6%), 1.4 at UAG (=3.5%/2.6%) and 6.0 at UGA (=14.4%/2.4%), respectively (Figure 2). The results of L123I, V and F were consistent with a previous report using yeast eRF1 (21,40).

Systematic mutagenesis clearly demonstrated that the position-123 residue of eRF1 plays a distinctive role in discriminating both adenine and guanine at the second nucleotide of stop codons, suggesting that position-123, and probably also its closely connected domains, is capable of taking on two structural modes. One is the A-mode that allows the decoding of adenine at the second base, thus resulting in UAA and UAG dual-specificity (prominent in the case of I and V), and the other is the G-mode that allows decoding of guanine at the second base, thus resulting in UGA specificity (prominent in the case of F, A, S, G and so on). L123P almost completely abolished activities at all stop codons, presumably due to structural defects, suggesting that the orientation of the flexible side chain as well as the main chain orientation of position-123 residues is important in the decoding of stop codons The cellular levels of those mutated proteins were comparable, as shown by western blot analysis (Supplementary Figure S2). For further analyses, we used a set of typical eRF1 mutants (L123I, V, F, A and S).

### Effect of heterogeneous combination of eRF1 and eRF3 on the codon-specificity profiles of the position-123 mutants of eRF1

**Figure 3.**
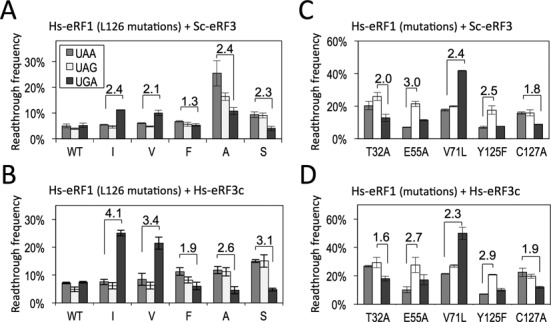
Readthrough assay of Hs-eRF1 mutants with Sc- or Hs-eRF3. (**A**) Readthrough frequencies of Hs-eRF1 wild type (WT) and position-123 mutants (L126 in human) with Sc-eRF3. The assay strains, S13-I01, S13-I03, S13-I05 and S13-I07, were transformed with the expression vector, either of wild type or position-123 mutants, of Hs-eRF1 on the p416GPD (URA3 marker) (Supplementary Table S4, rows ‘Sc-eRF3’). (**B**) Readthrough frequencies of Hs-eRF1 wild type (WT) and position-123 mutants (L126 in human) with Hs-eRF3c. The assay strains, SKY106, SKY107, SKY108 and SKY109, were transformed with the expression vector, either of wild type or position-123 mutants of Hs-eRF1 on the p415GPD (LEU2 marker) as well as with the expression vector of Hs-eRF3c on p416GPD (URA3 marker) (Supplementary Table S4, rows ‘Hs-eRF3c’). (**C**) Readthrough frequencies of Hs-eRF1 mutants of putative stop codon-binding residues (T32, E55 V71, Y125 and C127 of eRF1 in human numbering) with Sc-eRF3. The assay strains, S13-I01, S13-I03, S13-I05 and S13-I07, were transformed with the expression vector, either of wild type or mutants of Hs-eRF1 on p416GPD (URA3 marker) (Supplementary Table S5, rows ‘Sc-eRF3’; see Figure 3A and Supplementary Table S4 for wild type Hs-eRF1). (**D**) Readthrough frequencies of Hs-eRF1 mutants of putative stop codon-binding residues (T32, E55 V71, Y125 and C127 of eRF1 in human numbering) with Hs-eRF3c. The assay strains, SKY106, SKY107, SKY108 and SKY109, were transformed with the expression vector, either of wild type or mutants of Hs-eRF1 on p415GPD (LEU2 marker), as well as with the expression vector of Hs-eRF3c on p416GPD (URA3 marker) (Supplementary Table S5, rows ‘Hs-eRF3c’; see Figure 3B and Supplementary Table S4 for wild type Hs-eRF1). Readthrough frequencies are indicated as percentages, where 100% readthrough was based on the results of a UGG reporter gene, values are indicated as mean ± SD from three independent measurements. Fold differences between selected stop codons, rounded to the first decimal (Supplementary Tables S4 and S5), are indicated over the bars.

To test phylogenetic functional conservation of the mutational effects of the position-123 residue, human eRF1 (Hs-eRF1) was mutated at the same position, residue L126 (in human numbering, which corresponds to L123 of *S. cerevisiae* eRF1), and applied to the readthrough assay. As shown in Figure 3A and Supplementary Table S4, most of the Hs-eRF1 L126 mutants exhibited similar codon-specificity patterns to the corresponding L123 mutants of Sc-eRF1, although less markedly so.

Recently, we have demonstrated that a highly homologous eRF3 ortholog from a related fungus, *Pneumocystis carinii* (Pc-eRF3), cannot replace the endogenous eRF3 of *S. cerevisiae*, even though eRF3s from a wide range of species, from human to fission yeast, are believed to be essentially replaceable in budding yeast ((36) and references therein). Subsequent genetic analyses revealed that inadequate cooperation between heterogeneous Sc-eRF1 and Pc-eRF3 was compensated by mutations of conserved amino acid residues located at the interaction sites involved in eRF1–eRF3 complex formation, as well as at the putative ribosome-binding regions in eRF1 (36). This strongly suggested latent dysfunction among previous heterogeneous combinations of eRF1 and eRF3. These findings prompted us to test the effect of a heterogeneous combination of eRF1 and eRF3 on the apparent reduction of codon specificities of Hs-eRF1 L126 mutants.

The readthrough frequency of the Hs-eRF1 L126 mutants was monitored in a set of modified readthrough assay strains, SKY106, SKY107, SKY108 and SKY109, in which Hs-eRF3c (where ‘c’ denotes an eRF3 variant with truncation of the non-conserved N-terminal domain (41), hereafter referred to simply as Hs-eRF3c) is constitutively expressed from the expression vector, p416GPD, while endogenous Sc-eRF3 is downregulated upon addition of tetracycline (150 mM). Importantly, the readthrough frequency(ies) for the (sub-)specific codons was hardly affected, regardless of eRF3 species; however, defective stop codon recognition was enhanced with homogeneous combinations of Hs-eRF1 and Hs-eRF3 (Figure 3B and Supplementary Table S4). For example, in the case of Hs-eRF1 L126I, the ratio of readthrough frequency UAA/UGA was 4.1 with Hs-eRF3c and 2.4 with Sc-eRF3. These results demonstrated that the effect of the position-123 residue on stop codon discrimination is determined by the cooperativity of eRF1 with eRF3, suggesting the functional coupling of position-123 and eRF3.

### Comparison of the effect of the position-123 mutations with the putative stop codon-binding mutations of eRF1

Previously, we have predicted putative amino acid residues of eRF1 that may be involved in stop codon-binding (positions T32, E55, V71, Y125 and C127 of eRF1 in human numbering), based on the X-ray structure of an eRF1–eRF3 complex co-crystallized with an ATP molecule in domain N of eRF1. Moreover, mutation at those residues had altered the codon specificity of Hs-eRF1 (26). Our results had suggested that ATP binding potentially mimicked the binding of either the second or third base in the stop codons. As mentioned above, the position-123 residue directs its side chain differently from these neighboring residues (Supplementary Figure S1); therefore, distinctive functional roles in codon discrimination can be assumed.

In order to compare the roles of previous eRF1 mutations with that of the position-123 mutants, we examined the effects of the species combination of eRF1 and eRF3 by both types of the mutations. Unlike the Hs-eRF1 L126 mutants, intriguingly, the codon-specificity profiles of the putative stop codon-binding eRF1 mutations were barely affected by replacement of Sc-eRF3 with Hs-eRF3c (Figure 3C and D and Supplementary Table S5). This result suggested that those amino acid residues contribute to stop codon recognition in a way distinct from that by the position-123 residue, which involves eRF3 cooperativity.

### Altered codon specificities of the position-123 mutants demonstrated by the Dual-eRF1 complementation assay

**Figure 4.**
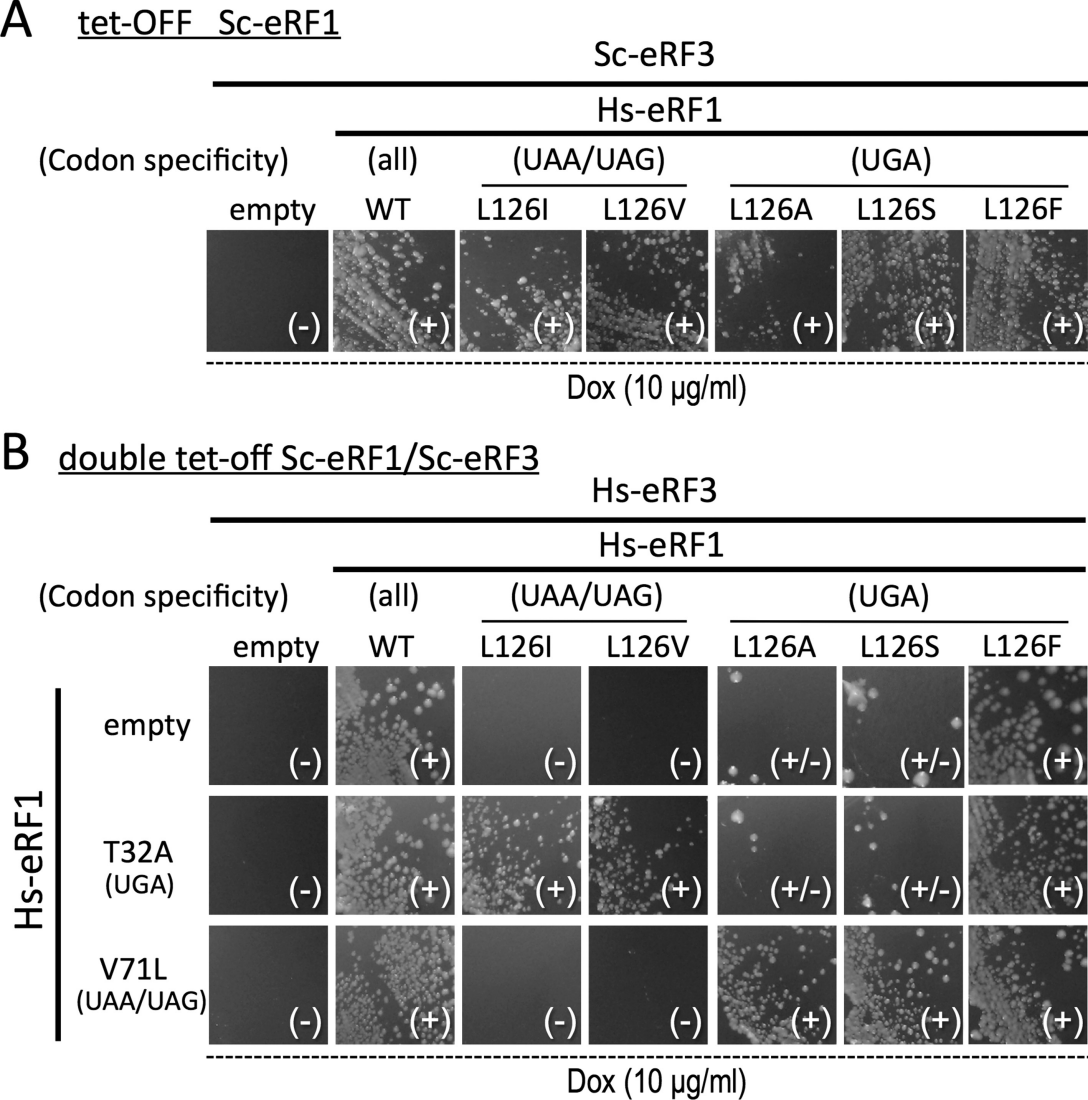
Growth complementation assays for the stop codon specificities of the Hs-eRF1 L126 mutants in the presence of Sc- or Hs-eRF3. (**A**) Growth complementation assay for single vector transformants of wild type (WT) and L126 mutants of Hs-eRF1 in the presence of heterologous Sc-eRF3 using Sc-eRF1 tet-off strain (S13-D09). Transformants of p416ADH (URA3 marker) (38) harboring each Hs-eRF1 variant grown under permissive conditions were streaked on the SC-Uracil plates containing the stable tetracycline analog (Dox) doxycycline (10 μg/ml) and their colony formation was monitored. (**B**) Growth complementation assay for dual vector transformants of L126 variants of Hs-eRF1 on p416ADH (URA3 marker) and Hs-eRF1 mutants T32A and V71L (26) on p415GPD (LEU2 marker) in the presence of homogeneous Hs-eRF3 on p414GPD (TRP1 marker) using the Sc-eRF1-Sc-eRF3 double tet-off strain (Y138) (38). Transformants grown under permissive conditions were streaked on the SC-Uracil-Leucine-Tryptophan plates containing the stable tetracycline analog doxycycline (Dox; 10 μg/ml) and their colony formation was monitored. Dox, doxycycline. Codon specificities are indicated: (all) omnipotent, i.e. UAG, UGA, UAA-specific (UAA/UAG) and (UGA) biased to be favorable to ‘UAA and UAG dual-specific’ and ‘UGA-specific’, respectively. Colony growth is indicated as (–) no growth at all, (±) very severe inhibition of growth with several revertant colonies and (+) normal growth.

A genetic analysis was carried out to further confirm the altered codon specificities of the position-123 mutations in Hs-eRF1 by *in vivo* growth complementation assays (26). First, a set of Hs-eRF1 L126 variants (wild type, L126I, L126V, L126A, L126S and L126F) was introduced into the eRF1 tet-OFF strain, in which the endogenous Sc-eRF3 is intact, and the cell growth was monitored at the non-permissive condition (10-μg/ml doxycycline). All of the transformants were viable, although Hs-eRF1 L126A grew slowly (Figure 4A), suggesting that the bias of Hs-eRF1-L126 mutations in codon specificity is less distinctive with heterogeneous Sc-eRF3 probably consistent with the readthrough efficiency result (Figure 3A).

Then, a modified version of dual-eRF1 growth complementation assay was employed (the Materials and Methods section). A set of Hs-eRF1 L126 variants (wild type, L126I, L126V, L126A, L126S and L126F) was co-introduced into the double tet-OFF strain with either the empty vector or the Hs-eRF1 T32A (UGA specific) or V71L (UAA and UAG dual-specific) mutants (26), as well as with homogeneous Hs-eRF3c, that is, three expression vectors in total. The cell growth of the transformants was monitored under non-permissive conditions (10-μg/ml doxycycline). As shown in Figure 4B, Hs-eRF1 L126I and L126V transformants hardly grew when combined with the empty vector and Hs-eRF1 V71L, but these transformants grew well when co-expressed with Hs-eRF1 T32A that has complementary specificity (Figure 3B). Similarly, L126A and L126S transformants only grew normally when they were co-expressed with Hs-eRF1 V71L. The only exception was the Hs-eRF1 L126F mutant, which exhibited cell growth, as the activity was sufficient, as indicated by readthrough assays (Figure 2). Consequently, the results of the modified Dual-eRF1 assay strengthened the biological significances of the position-123 residue of eRF1 as well as its proper functional coupling with eRF3 in stop codon discrimination.

### Effect of paromomycin on the codon specificity of position-123 mutants of eRF1

Since the position-123 of eRF1 is located close to the putative tRNA-anticodon mimicking site distant from eRF3 in the complex structures (26,33), functional coupling is likely to be achieved indirectly rather than by direct physical interactions. To obtain a clue to the coupling between physically separated regions in eRF1 and eRF3, we next examined the effect of paromomycin on the codon recognition profiles of position-123 mutants of eRF1. Paromomycin is an aminoglycoside antibiotic that specifically binds to the decoding site rRNA residues and forces nucleotides A1492 and A1493 of 16S rRNA (A1755 and A1756 of yeast 18S rRNA) to flip out in a manner similar to that which occurs in the presence of cognate tRNA and mRNA, resulting in uncoupling of tRNA and its carrier GTPase (EF-Tu/eEF1A), and inducing severe defects in decoding fidelity (42). Paromomycin was also shown to act on the eukaryotic ribosome, although the effective concentration for cell growth inhibition was reported to be much higher in yeast, due to differences in non-conserved nucleotides surrounding A1755 and A1756 (43).

**Figure 5.**
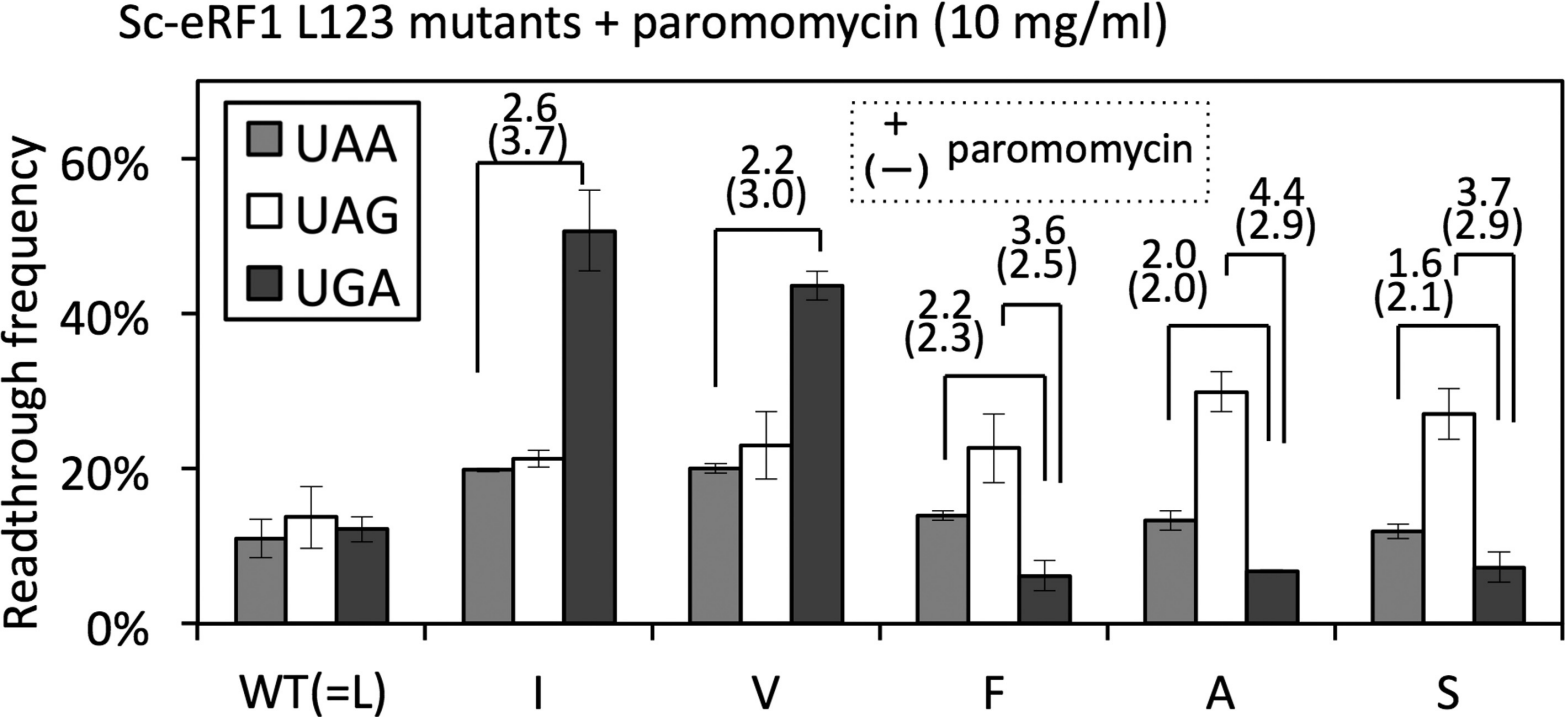
Readthrough assay of Sc-eRF1 wild type (WT) and L123 mutants with paromomycin. Readthrough frequencies of Sc-eRF1 WT and L123 mutants in the presence of paromomycin (10 mg/ml). The assay strains, SKY81, SKY82, SKY83 and SKY84, were transformed with the expression vector, either of the wild type or position-123 mutants, of Sc-eRF1 on the p415GPD (LEU2 marker; Supplementary Table S6, rows ‘+paromomycin’). Paromomycin was added to the medium at the timing of temperature shift and refreshing. Wild type and mutant Sc-eRF1 readthrough frequencies (Supplementary Table S6) are indicated by percentage, where 100% readthrough was based on the results of a UGG reporter gene; values are indicated as mean ± SD from three independent measurements. Fold differences between selected stop codons, rounded to the first decimal place, are indicated over the bars with the values in the absence of paromomycin in the parentheses below (Supplementary Table S6).

The ratio of readthrough frequency for UAA- and UAG-specific eRF1s, L123I and L123V, became less marked in the presence of paromomycin (Figure 5); e.g. for Sc-eRF1 L123I, UGA/UAA ratio was 2.6 (50.7/19.8%) in the presence of paromomycin, but was 3.7 in the absence of paromomycin. This might, in part, reflect specific inhibition of the eRF3-coupled decoding function of the position-123 residue by paromomycin. However, upon addition of paromomycin, the readthrough frequency in wild-type Sc-eRF1 transformants increased equally for all three stop codons (Figure 5 and Supplementary Table S6), i.e. paromomycin raised the basal levels of readthrough efficiency about 4-fold. Thus, the codon specificity ratios between the data sets in the presence and absence of paromomycin might not simply be applicable to the analysis of Hs-eRF1 L126 mutants with different eRF3s (Figure 3).

Nevertheless, it is noteworthy that, paromomycin increased the readthrough frequency of UGA-specific Sc-eRF1s (L123F, L123A, L123S) prominently at the UAG stop codon than other stop codons. For instance, UAA/UGA ratios of readthrough frequencies for Sc-eRF1 L123F were =2.2 in the presence of paromomycin, 2.3 in its absence, and that of UAG/UGA were 3.6 in the presence of paromomycin and 2.47 in its absence. Collectively paromomycin affected Sc-eRF1 L123 mutants in differential ways; (1) reduction of the bias in UAA/UAG-specific eRF1 mutants, and increase of the bias in UGA-specific eRF1s for UAG codon. The effect of paromomycin on position-123 mutants of eRF1 is discussed in the next section.

## DISCUSSION

In this report, we have studied the conserved residue at position 123 of eRF1 by functional analyses in yeast. According to the result of systematic mutagenesis (Figure 2), we can postulate a fundamental molecular mechanism for the second base discrimination by the position-123 residue of eRF1 based on the side-chain properties. We found that truncation of the side chain by mutating it to glycine (G), alanine (A) or serine (S) made eRF1 less efficiently recognize UAA and UAG codons, suggesting that the basic structure of eRF1 for codon discrimination preferably allows UGA recognition in the absence of side-chain interaction at this position. Extension of the side chain, by mutating position-123 to cysteine (C), threonine (T), methionine (M) or leucine (L) (the wild type residue) endows eRF1 with omnipotent stop codon recognition. Importantly, replacement of the residue with serine (S) or cysteine (C) indicated that there was a clear size boundary between UGA-specificity and omnipotence lies between, depending on whether oxygen and/or sulfur was attached to the beta carbon. A similar size-dependency was seen when the residue was mutated to aspartic acid (D)/asparagine (N) or to glutamic acid (E)/glutamate (Q). Furthermore, mutation of the residue to a branched amino acid at the beta carbon, i.e. isoleucine (I) or valine (V), led to UAA and UAG codon specificity, again pointing out the importance of the precise physical properties of the side chain and the size boundary. Although further structural and biochemical studies are required to test this idea in more detail, we presume that this is why leucine, rather than other amino acid residues with similar properties, is conserved at position-123 among omnipotent eRF1s of species using the standard genetic code (Figure 1B). Additionally, further side-chain expansion with amino acids with longer side chains (such as lysine [K] and arginine [R]) or with bulky aromatic moieties (such as phenylalanine [F], tyrosine [Y], histidine [H] and tryptophan [W]) enhanced the recognition of the UGA codon. This may suggest that the conformation of the position-123 residue is rather flexible, and sufficiently so to allow a range of sizes.

Previously, it has been reported that eRF1 provokes differential kinetics of GTP hydrolysis by eRF3 for UAA and UAG (fast) versus UGA (slow) codons (30). The authors of that report proposed that a putative conformational change occurs at the local domain of eRF1 in response to each of these stop codons, with the most marked change occurring in response to UGA. This observation is in good agreement with our finding, since domain N favors UGA in the absence of extended position-123 side chains (Figure 2), and the function of the postion-123 is strongly coupled with the compatibility of eRF1 with eRF3 (Figures 3 and 4).

To study the functional conservation of the position-123 residue, the activities of Hs-eRF1 mutants at the corresponding residue, L126 (in human eRF1 numbering), were analyzed. Hs-eRF1 L126 mutants exhibited similar codon-specificities as Sc-eRF1 L123 mutants, suggesting the functional conservation of L126, which is consistent with the results of studies on ciliate eRF1s (21,22). However, the trends for codon-specificities in Hs-eRF1 L126 mutants in *S. cerevisiae* cells, which express Sc-eRF3, were more moderate than those of Sc-eRF1 L123 mutants (Figure 3A) and, interestingly, these moderate characteristics could be regained by co-expression of homogeneous Hs-eRF3c instead of Sc-eRF3 (Figure 3B). In contrast, other previously reported mutations were unaffected by eRF3 replacement. The Dual-eRF1 assay results further verified this phenomenon (Figure 4B). These results suggested a functional coupling of eRF1 and eRF3 that occurs specifically through the position-123 residue of eRF1.

Addition of a decoding-influencing antibiotic, paromomycin, also significantly altered the specificity of Sc-eRF1 L123 mutants (Figure 5). Paromomycin reduced a codon-specificity bias for UAA and UAG dual-specific Sc-eRF1 L123 mutants (Figure 5). The inhibitory mode of action of paromomycin on eubacterial translation elongation involves the flipping-out of A1492 and A1493 (*Escherichia coli* numbering; A1755 and A1756 of the 18S rRNA in *S. cerevisiae*), which induces mandatory GTP hydrolysis by EF-Tu without cognate codon–anticodon pairing (44). Therefore, these results suggested that the side chain of the position-123 residue of eRF1 functions in a manner that is closely related to the target site of paromomycin, i.e. A1755 and A1756 of the 18S rRNA in *S. cerevisiae*, to induce conformational changes in the ribosome that allows stop codon decoding.

**Figure 6.**
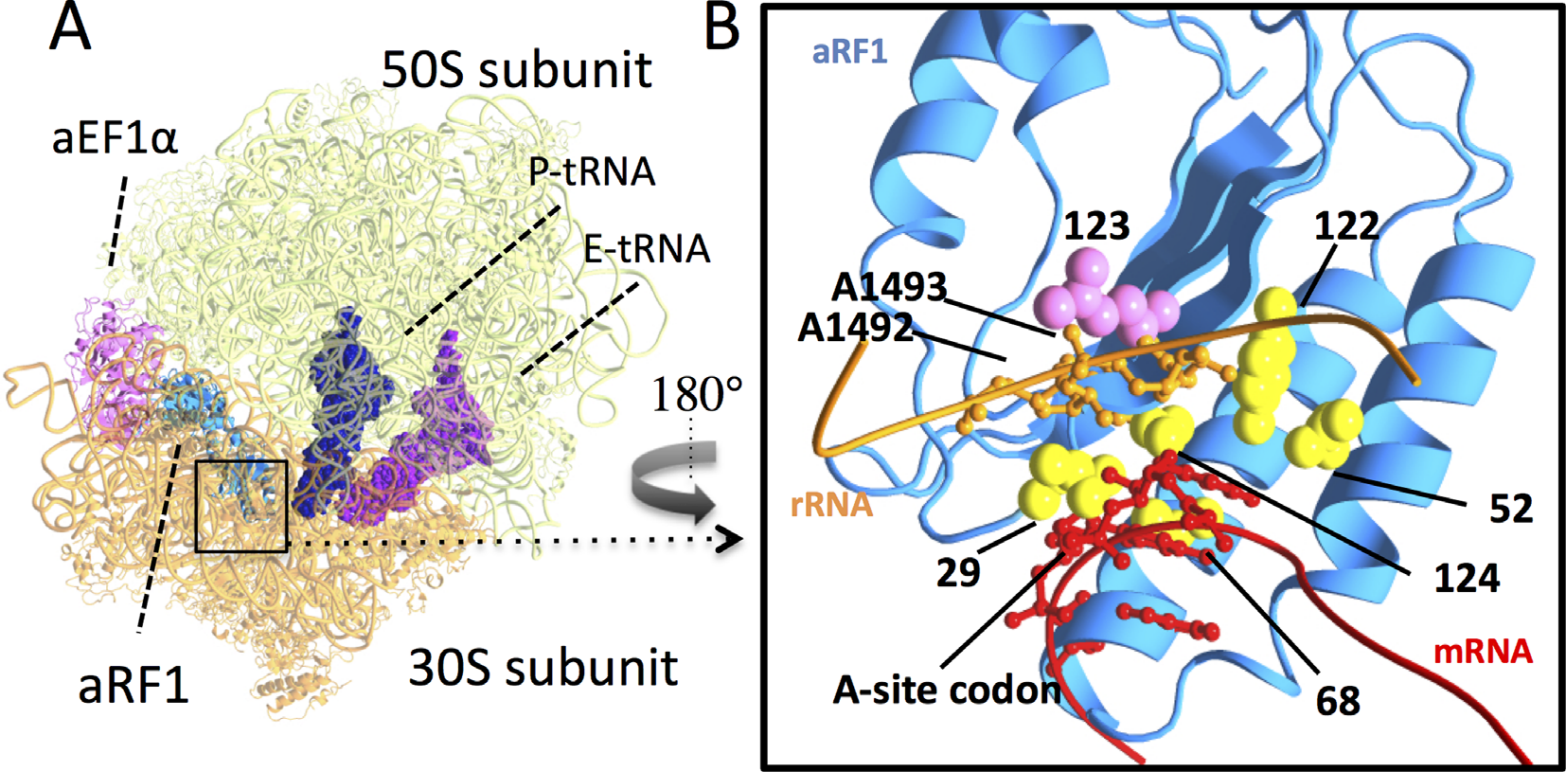
Putative docking model of the ribosome and eRF1 in a quasi-A/T state. (**A**) Overall structure. Molecules in the docking model are indicated: aRF1 (light blue), aEF1A (pink), P-site tRNA (dark blue), E-site tRNA (purple), 30S subunits (orange) and 50S subunits (light green) (PDB ID: 3VMF, 2WRO). (**B**) Detailed view of the decoding center (in the rectangle in (A), opposite view); Domain N of aRF1 (light blue), position-123 (purple) and putative stop codon-binding residues, T29, E52, V68, Y122 and C124, in *Saccharomyces cerevisiae* numbering (yellow), rRNA (residues 1488–1496, *in Escherichia coli* numbering) (orange) and mRNA (codon triplets and each of the two bases in the 5′ and 3′ flanking regions) (red). eRF1 residues are shown with *S. cerevisiae* numbering. Side chains of aRF1 are shown in a sphere model. A1492 and A1493 of rRNA and A-site codons are shown using a ball and stick model. Structural models were rendered by MolFeat version 3.5 (Fiatlux, Tokyo, Japan).

To address this issue, a docking model of aRF1–aEF1A–GTP (PDB ID: 3VMF) was constructed using the high-resolution bacterial A/T state ribosome (tRNA–EF-Tu–GTP–ribosome, ID: 2WRN, 2WRO) (45), as we reported previously (17,33); this model was based on the conserved ribosomal decoding structure among all phylogenetic domains (11,15). In our model, intriguingly, the protruding side chain of L123 was in close contact with A1492 and A1493 at the edge of helix 44 of the 16S rRNA in the decoding site, whereas other side chains that have been reported to be involved in stop codon decoding are in close contact with the A site codon bases of the mRNA (Figure 6). It has been hypothesized that the decoding of sense and stop codons in eukaryotes and archaea share common GTPase-dependent mechanisms, based on the striking molecular mimicry between tRNA and eRF1 (aRF1) in complex with the translational GTPase (16). Thus, it is tempting to assume a putative contact between the position-123 side chain and rRNA moieties, which mimics the contact between tRNAs and rRNA for transmission of codon binding to the GTPase center, as a molecular explanation for the functional coupling of the position-L123 residue and eRF3 (Figures 3 and 4). The distorted orientation of A1755 and A1756 by paromomycin presumably perturbs the correct interaction with position-123 of eRF1, leading to less marked codon specificities of UAA- and UAG-specific mutants as in the heterogeneous combinations of eRF1–eRF3.

In eubacterial stop codon decoding by RF1 and RF2, in which GTP hydrolysis is not involved, paromomycin is thought to inhibit cognate stop codon recognition by competing with RF1 for the local conformational rearrangement of the decoding center (46). Therefore, it is possible that position-123 of eRF1 may have some effects analogous to eubacterial stop codon decoding, which could be affected by paromomycin. The UGA-specific mutants (L123F, L123A and L123S) exhibited significantly increased readthrough frequency at UAG in the presence of paromomycin. This is distinctive from the results seen with co-expression of different eRF3s (Figure 3A and B). These observations suggest that the preferential effect of paromomycin on eRF1 mutants could arise from local conformation rearrangements in the paromomycin-bound decoding center (Figure 6B).

Consequently, as a working hypothesis, the following scheme could be used to explain the role of the position-123 residue of eRF1 in decoding stop codons. First, after initial binding of the eRF1–eRF3–GTP complex to the ribosome, close local interaction of the position-123 side chain with the ribosomal A1755 and 1756 residues, as well as switching of the domain N conformation (A-mode or G-mode), occurs in response to recognition of stop codon at the A-site of ribosome (quasi-A/T state). Subsequently, the interaction between position-123 and A1755 and A1756 is likely to induce a conformational change in the ribosome that transmits cognate stop codon recognition in a manner similar to that occurs between tRNA and eEF1A /EF-Tu for sense codon decoding.

Several amino acid residues for stop codon discrimination in the putative anticodon mimicking domains of eRF1 have been proposed to date (8,18,24,26). Many of them are expected to form codon-binding pockets. Recently Blanchet *et al*. reported local conformational changes of the codon-binding pocket of eRF1 by nuclear magnetic resonance structural study of systematic mutations in the codon-binding pockets (47). However, those putative codon contact sites *per se* would not fully explain the mechanism for codon discrimination patterns of eRF1. Our genetic results suggested a novel role, which is distinct from direct codon binding, of the amino acid residue in the anticodon-mimicking domains of eRF1. The critical residue, position-123 of eRF1, could function in stop codon discrimination through interaction with the ribosomal decoding sites, potentially in a manner similar to that for the coupling of carrier GTPase with tRNA. To elucidate the whole process of translation termination in eukaryotes, further structural, biochemical and genetic analyses of the putative quasi-A/A state of eRF1 on the ribosome, after precise codon recognition in the quasi-A/T state, are required.

## SUPPLEMENTARY DATA

Supplementary Data are available at NAR Online.

SUPPLEMENTARY DATA
