# Supplementary material for: Genetic analysis of L123 of the tRNA-mimicking eukaryote release factor eRF1, an amino acid residue critical for discrimination of stop codons
Source: Nucleic Acids Res. 2015 Apr 20;43(9):4591–601. doi: 10.1093/nar/gkv376 (PMC4482090; doi:10.1093/nar/gkv376)
Supplement: SUPPLEMENTARY DATA [file supp_43_9_4591__index.html]

Genetic analysis of L123 of the tRNA-mimicking eukaryote release factor eRF1, an amino acid residue critical for discrimination of stop codons — Genetic analysis of L123 of the tRNA-mimicking eukaryote release factor eRF1, an amino acid residue critical for discrimination of stop codons — SUPPLEMENTARY DATA 

# Genetic analysis of L123 of the tRNA-mimicking eukaryote release factor eRF1, an amino acid residue critical for discrimination of stop codons

## SUPPLEMENTARY DATA

**Files in this Data Supplement:**

- SUPPLEMENTARY DATA
