# Supplementary material for: Genetic analysis of L123 of the tRNA-mimicking eukaryote release factor eRF1, an amino acid residue critical for discrimination of stop codons
Source: Nucleic Acids Res. 2015 Apr 20;43(9):4591–601. doi: 10.1093/nar/gkv376 (PMC4482090; doi:10.1093/nar/gkv376)

SUPPLEMENTARY

Genetic analysis of L123 of the tRNA-mimicking eukaryote release factor eRF1, an amino acid residue critical for discrimination of stop codons

Kazuki Saito[1] and Koichi Ito[1]*

[1]Department of Medical Genome Sciences, Graduate School of Frontier Sciences, The University of Tokyo, Kashiwa-city, Chiba 277-8562, Japan

* To whom correspondence should be addressed. Tel: +81 4 7136 3600; Fax: +81 4 7136 3601; Email: itokoichi@k.u-tokyo.ac.jp

SUPPLEMENTARY TABLE AND FIGURES LEGENDS

Table S1. Yeast strains used in this study.

Table S2. Primers used for construction of yeast expression plasmids.

Table S3. Readthrough frequencies of comprehensive Sc-eRF1 L123 mutants.

Table S4. Summary of the readthrough frequencies of Hs-eRF1 L126 mutants in various conditions.

Table S5. Readthrough frequencies of Hs-eRF1 mutants other than L126.

Table S6. Readthrough frequencies of selected Sc-eRF1 L123 mutants.

Figure S1. Structural presentation of the position-123 residues in the various crystal structures of eRF1 (aRF1). The domain N of various eRF1s with relevant amino acid residues are shown using the previously reported apo- and complexed forms of eRF1/aRF1 (apo-form of *Aeropyrum pernix* aRF1 (pdb id:3AGK), aRF1 in an aEF1A-bound complex (pdb id:3VMF), apo-form of *Homo sapiens* eRF1 [pdb id: 1DT9], human eRF1 in an eRF3-bound complex [pdb id: 3E1Y]). In all structures, the position-123 residue is shown in purple, and residues that contain putative stop codon-binding pockets (T29, E52, V68, Y122, and C124, in *Saccharomyces cerevisiae* numbering) are shown in yellow. Structural models were rendered by MolFeat version 3.5 (Fiatlux, Tokyo).

Figure S2. Western blot analysis of cellular protein levels of Myc-tagged Sc-eRF1 wild type (WT) and L123 mutants. Protein expression levels of Myc-tagged Sc-eRF1 variants in the assay strain were monitored by western blot analysis using an anti-myc antibody (Sigma–Aldrich, St Louis, MO, USA). Cellular levels of endogenous PGK1 were monitored as loading control, using an anti-PGK antibody (Invitrogen, Carlsbad, CA, USA). Yeast transformant cells were collected and precipitated with 10% trichloroacetic acid, suspended in SDS sample buffer, neutralized with 5 N NaOH solution, and vigorously mixed with glass beads on the FastPrep 24 instrument (MP Biomedicals, Santa Ana, CA, USA). Proteins in the cell extracts were separated by SDS-PAGE, transferred to polyvinylidene fluoride membranes, and detected using the appropriate antibodies and an enhanced chemiluminescence western blot detection system (GE Healthcare, Little Chalfont, UK).

Table S1
Yeast strains used in this study

Name	Genotype	Reference
S13-I01	*MATα ura3-1 ade2-1 leu2-3,112 sal4-2* (eRF1ts) *HO::kanMX-Rluc*-[UAA]-*Luc2*	(49)
S13-I03	*MATα ura3-1 ade2-1 leu2-3,112 sal4-2* (eRF1ts) *HO::kanMX-Rluc*-[UAG]-*Luc2*	(49)
S13-I05	*MATα ura3-1 ade2-1 leu2-3,112 sal4-2* (eRF1ts) *HO::kanMX-Rluc*-[UGA]-*Luc2*	(49)
S13-I07	*MATα ura3-1 ade2-1 leu2-3,112 sal4-2* (eRF1ts) *HO::kanMX-Rluc*-[UGG]-*Luc2*	(49)
S13-D09	*MATα ade2Δ::hisG his3Δ200 leu2Δ0 lys2Δ0 met15Δ0 trp1Δ63 ura3Δ0 tTA-tetO (*YBR143C/*SUP45)::hphMX4*	(26)
Y138	*MATα ade2Δ::hisG his3Δ200 leu2Δ0 lys2Δ0 met15Δ0 trp1Δ63 ura3Δ0 SUP35::HIS3 HO::tTA-tetO (*YDR172W/*SUP35):: kanMX4 tTA-tetO (*YBR143C/*SUP45)::hphMX4*	(36)
SKY81	*MATα ura3-1 ade2-1 leu2-3,112 sal4-2* (eRF1ts) *HO::CaURA3-R luc*-[UAA]-*Luc2*	This work
SKY82	*MATα ura3-1 ade2-1 leu2-3,112 sal4-2* (eRF1ts) *HO::CaURA3-Rluc*-[UAG]-*Luc2*	This work
SKY83	*MATα ura3-1 ade2-1 leu2-3,112 sal4-2* (eRF1ts) *HO::CaURA3-Rluc*-[UGA]-*Luc2*	This work
SKY84	*MATα ura3-1 ade2-1 leu2-3,112 sal4-2* (eRF1ts) *HO::CaURA3-Rluc*-[UGG]-*Luc2*	This work
SKY106	*MATα ura3-1 ade2-1 leu2-3,112 sal4-2* (eRF1ts) *HO::kanMX-Rluc*-[UAA]-*Luc2* *tTA-tetO-SUP35 (*eRF3$^+$*)*	This work
SKY107	*MATα ura3-1 ade2-1 leu2-3,112 sal4-2* (eRF1ts) *HO::kanMX-Rluc*-[UAG]-*Luc2* *tTA-tetO-SUP35 (*eRF3$^+$*)*	This work
SKY108	*MATα ura3-1 ade2-1 leu2-3,112 sal4-2* (eRF1ts) *HO::kanMX-Rluc*-[UGA]-*Luc2* *tTA-tetO-SUP35 (*eRF3$^+$*)*	This work
SKY109	*MATα ura3-1 ade2-1 leu2-3,112 sal4-2* (eRF1ts) *HO::kanMX-Rluc*-[UGG]-*Luc2* *tTA-tetO-SUP35 (*eRF3$^+$*)*	This work

Table S2
Primers used for construction of yeast expression plasmids

Name of Primers	Sequences	Restriction site (Underlined in sequence)
Sc-eRF1N [*1]	5'-GGGATCCATATGGATAACGAGGTTGAAAAA-3'	*Bam*HI
Sc-eRF1C [*1]	5'-GGGTCGACTTAAATGAAATCATAGTCAGATCC-3'	*Sal*I
Hs-eRF1N	5'-GGAATTCCATATGGCGGACGACCCCAGTGC-3'	*Eco*RI
Hs-eRF1C	5'-GGGTCGACCTAGTAGTCATCAAGGTCAAAAAATTC-3'	*Sal*I
Hs-eRF3cN	5'-GGGAATTCCATATGATGGAGGAGGAAGAGGA-3'	*Eco*RI
Hs-eRF3C	5'-GGGTCGACTTAGTCTTTCTCTGGAACCAG-3'	*Sal*I

*1. "N" and "C" denotes the N- and C-terminal position of the open reading frame, respectively.

Table S3
Readthrough frequencies of comprehensive Sc-eRF1 L123 mutants.

Sc-eRF1 Mutation	Readthrough frequency [*1]		
	UAA	UAG	UGA
Vector (p416GPD)	27.2%	24.6%	24.1%
WT	2.6%	2.6%	2.4%
L123I	3.9% (1.5) [*2]	3.5% (1.4)	14.4% (6.0)
L123V	2.6%	2.6%	7.7%
L123F	3.4%	3.7%	1.5%
L123A	4.8%	7.0%	2.4%
L123S	4.9%	6.7%	2.3%
L123T	3.3%	3.8%	3.9%
L123C	3.4%	3.8%	3.1%
L123M	3.1%	3.0%	3.1%
L123G	7.0%	7.2%	2.2%
L123D	7.5%	5.0%	3.7%
L123E	5.7%	4.7%	6.4%
L123N	5.0%	4.1%	2.6%
L123Q	4.4%	5.4%	3.4%
L123R	2.5%	3.2%	1.3%
L123K	2.7%	3.3%	1.6%
L123H	4.0%	3.1%	1.9%
L123Y	3.2%	3.4%	1.9%
L123W	4.0%	2.9%	3.0%
L123P	28.8%	27.7%	21.9%

*1. Readthrough frequencies are indicated as mean from three independent measurements. The assay strains S13-I01, S13-I03, S13-I05, and S13-I07 (Table S5) were used.

*2 Fold-changes to the wild type values are rounded to the first decimal place are shown in parentheses.

Table S4

Summary of the readthrough frequencies of Hs-eRF1 L126 mutants under various conditions

Hs-eRF1 Mutation [*1]	Additional condition [*2]	Readthrough frequency [*3]			Fold-change [*6]		
		UAA	UAG	UGA	UAA	UAG	UGA
WT	Sc-eRF3[*4]	5.0%	3.9%	5.2%	1.3	(1)	1.3
	Hs-eRF3c[*5]	7.2%	4.9%	7.4%	1.5	(1)	1.5
L126I	Sc-eRF3	5.5%	4.6%	11.2%	1.2	(1)	**2.4**[*7]
	Hs-eRF3c	7.6%	6.2%	25.1%	1.2	(1)	**4.1**
L126V	Sc-eRF3	6.1%	4.8%	10.0%	1.3	(1)	**2.1**
	Hs-eRF3c	8.5%	6.2%	21.5%	1.4	(1)	**3.4**
L126F	Sc-eRF3	6.8%	5.6%	5.3%	**1.3**	1.1	(1)
	Hs-eRF3c	11.3%	8.2%	6.1%	**1.9**	1.4	(1)
L126A	Sc-eRF3	25.4%	16.3%	10.8%	**2.4**	1.5	(1)
	Hs-eRF3c	11.8%	11.2%	4.5%	**2.6**	2.5	(1)
L126S	Sc-eRF3	9.3%	9.1%	4.0%	**2.3**	2.3	(1)
	Hs-eRF3c	15.0%	15.0%	4.8%	**3.1**	3.1	(1)

*1. Wild type and mutant Hs-eRF1s were expressed from the p416GPD (URA3 marker) expression vector in the case of "with Sc-eRF3" or from the p415GPD (LEU2 marker) in the case of "Hs-eRF3c" and "+paromomycin (with Hs-eRF3c)".

*2. The assay strains S13-I01, I03, I05, I07 (for additional condition; Sc-eRF3c), SKY106-109 (for additional condition; Hs-eRF3c), SKY143-146 (for additional condition; paromomycin) were used.

*3. Readthrough frequencies are indicated as the mean from three independent measurements.

*4. Sc-eRF3 is expressed from the endogenous wild type SUP35 gene in the strains S13-I01, I03, I05, and I07.

*5. Hs-eRF3 is expressed from the p416GPD (URA3 marker) expression vector. The endogenous Sc-eRF3 is downregulated in the presence of tetracycline (150 mM).

*6 Fold-changes to selected stop codon, shown as (1), are rounded to the first decimal place.

*7. Values cited in Fig.3AB are shown in boldface.

Table S5
Readthrough frequencies of Hs-eRF1 mutants other than L126

Hs-eRF1 Mutation [*1]	Additional condition [*2]	Readthrough frequency [*3]			Fold-change [*6]		
		UAA	UAG	UGA	UAA	UAG	UGA
T32A	Sc-eRF3 [*4]	20.4%	26.0%	12.8%	1.6	**2.0**	(1)
	Hs-eRF3c [*5]	26.7%	29.3%	18.0%	1.5	**1.6**	(1)
E55A	Sc-eRF3	7.1%	21.4%	11.4%	(1)	**3.0**	1.6
	Hs-eRF3c	10.1%	27.6%	17.3%	(1)	**2.7**	1.7
V71L	Sc-eRF3	17.7%	19.9%	41.9%	(1)	1.1	**2.4**
	Hs-eRF3c	21.4%	27.0%	49.9%	(1)	1.3	**2.3**
Y125F	Sc-eRF3	7.0%	17.6%	7.6%	(1)	**2.5**	1.1
	Hs-eRF3c	7.1%	20.8%	10.2%	(1)	**2.9**	1.4
C127A	Sc-eRF3	15.8%	15.9%	8.8%	**1.8**	1.8	(1)
	Hs-eRF3c	22.4%	19.7%	12.0%	**1.9**	1.6	(1)

*1. Wild type and mutant Hs-eRF1s were expressed from the p416GPD (URA3 marker) expression vector in the case of "Sc-eRF3" or from the p415GPD (LEU2 marker) in the case of "Hs-eRF3c" and "+paromomycin (with Hs-eRF3c)". See Table S2 for wild type Hs-eRF1
*2. The assay strains S13-I01, I03, I05, I07 (for additional condition; Sc-eRF3c), SKY106-109 (for additional condition; Hs-eRF3c), SKY143-146 (for additional condition; paromomycin) were used.
*3. Readthrough frequencies are indicated as the mean from three independent measurements.
*4. Sc-eRF3 is expressed from the endogenous wild type SUP35 gene in the strains S13-I01, I03, I05, and I07.
*5. Hs-eRF3 is expressed from the p416GPD (URA3 marker) expression vector. The endogenous Sc-eRF3 is downregulated in the presence of tetracycline (150 mM).
*6 Fold-changes to selected stop codon, shown as (1), are rounded to the first decimal place.
*7. Values cited in Fig.3CD are shown in boldface.

Table S6
Readthrough frequencies of selected Sc-eRF1 L123 mutants

eRF1 Mutation *1	Additional condition*1	Readthrough frequency*2			Fold-change *3		
		UAA	UAG	UGA	UAA	UAG	UGA
WT	(-paromomycin)	(2.6%)	(2.6%)	(2.4%)	(1)	1.0	0.9
	+paromomycin	11.0%	13.7%	12.2%	(1)	1.3	1.1
L123I	(-paromomycin)	(3.9%)	(3.5%)	(14.4%)	1.1	(1)	**3.7**
	+paromomycin	19.8%	21.3%	50.7%	(1)	1.1	**2.6**
L123V	(-paromomycin)	(2.6%)	(2.6%)	(7.7%)	(1)	1.0	**3.0**
	+paromomycin	20.0%	23.0%	43.6%	(1)	1.2	**2.2**
L123F	(-paromomycin)	(3.4%)	(3.7%)	(1.5%)	2.3	**2.5**	(1)
	+paromomycin	13.9%	22.6%	6.2%	2.2	**3.6**	(1)
L123A	(-paromomycin)	(4.8%)	(7.0%)	(2.4%)	2.0	**2.9**	(1)
	+paromomycin	13.3%	29.9%	6.8%	2.0	**4.4**	(1)
L123S	(-paromomycin)	(4.9%)	(6.7%)	(2.3%)	2.1	**2.9**	(1)
	+paromomycin	11.9%	27.1%	7.3%	1.6	**3.7**	(1)

1. "-paromomycin": The readthrough frequency data (parenthesized)* was cited from Table S1. "-paromomycin": The assay strains SKY81, SKY82, SKY83 and SKY84 harboring either the wild type or mutant Ss-eRF1 expression vectors based on the p415GPD (LEU2 marker) in the presence of paromomycin (+paromomycin [10 mg/ml]).
*2. Readthrough frequencies are indicated as the mean from three independent measurements.
*3 Fold-changes to selected stop codon, shown as (1), are rounded to the first decimal place.
*4. Values cited in Fig.3 are shown in boldface.

Figure S1

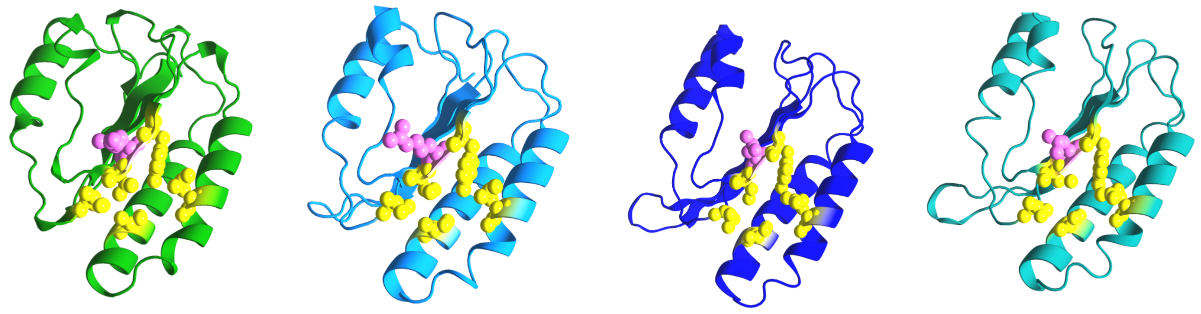

apo-
A. pernix aRF1
(3AGK)

aEF1α bound
A. pernix aRF1
(3VMF)

apo-
Human eRF1
(1DT9)

eRF3 bound
human eRF1
(3E1Y)

Figure S2

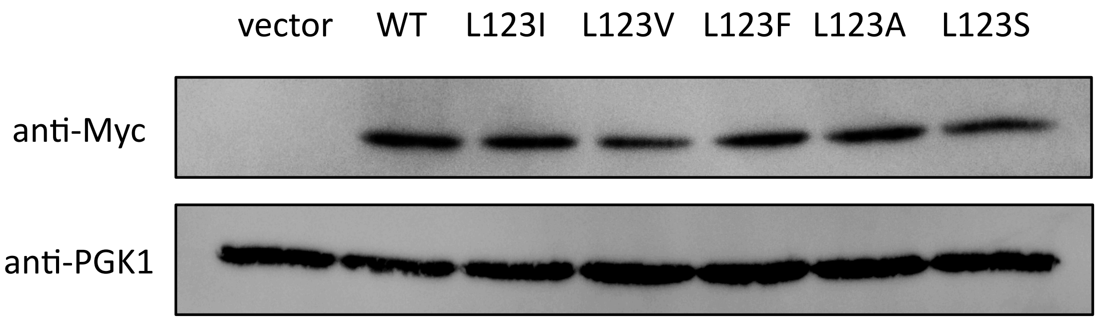

Supplement: SUPPLEMENTARY DATA [file supp_gkv376_nar-00601-z-2015-File008.pdf]
